# Anti‐depression molecular mechanism elucidation of the phytochemicals in edible flower of *Hemerocallis citrina* Baroni

**DOI:** 10.1002/fsn3.4446

**Published:** 2024-10-31

**Authors:** Ruohan Zhao, Jinhai Luo, Sookja Kim Chung, Baojun Xu

**Affiliations:** ^1^ Food Science and Technology Program, Department of Life Sciences BNU‐HKBU United International College Zhuhai Guangdong China; ^2^ Faculty of Medicine Macau University of Science and Technology Macau China

**Keywords:** core targets, depression, *Hemerocallis citrina* Baroni, molecular docking, network pharmacology

## Abstract

The edible flower of *Hemerocallis citrina* Baroni, commonly known as “Huang Huacai” in China, has anti‐depressant effects. However, targets and molecular mechanisms of *Hemerocallis citrina* Baroni edible flowers (HEF) in depression treatment are still unclear. The potential anti‐depression targets in HEF were identified by the intersecting results from typical drug databases. The network construction and Kyoto Encyclopedia of Genes and Genomes (KEGG) and Gene Ontology (GO) enrichment analysis were carried out for core targets. The molecular docking was conducted to predict the binding affinity between the active components and the central targets. The intersecting results indicated that there were 24 active components in HEF, with 449 anti‐depression targets identified. After screening through degree centrality (DC), betweenness centrality (BC), and closeness centrality (CC), 166 core targets were determined. Tumor protein 53 (TP53) and interleukin 6 (IL‐6) had the highest degree values. The results of GO enrichment analysis associated with anti‐depression revealed that the biological processes were negative regulation of osteoclast differentiation and positive regulation of phosphorus metabolic process. KEGG enrichment analysis results revealed that pathways, such as the phosphatidylinositol 3‑kinase‐protein kinase B (PI3K‐Akt) signaling pathway and mitogen‐activated protein kinase (MAPK) signaling pathway, were primarily associated with anti‐depression. Molecular docking results indicated that the top 10 active ingredients in HEF could bind to the central targets. This study applied network pharmacology to unveil the potential anti‐depressive mechanisms of HEF, providing a theoretical basis for further exploration of the effective components in *H*. *citrina* edible flower parts.

## Graphical abstract

This study applied network pharmacology to unveil the potential anti‐depressive mechanisms of *Hemerocallis*. *citrina* Baroni edible flowers (HEF), providing a theoretical basis for further exploration of the effective components in *H*. *citrina* edible flower parts.

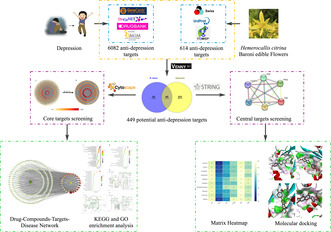

## 1 INTRODUCTION

Depression is a common illness that occurs across all genders, ages, social backgrounds, and even in animals. According to predictions by the World Health Organization (2017), by the year 2030, depression will be expected to become the disease with the highest economic burden worldwide. The primary clinical manifestations of depression include low mood, feelings of guilt, loss of interest, increased or decreased appetite, sleep disturbances, and difficulty concentrating, which have severely threatened human health (Perić et al., 2021). Additionally, the occurrence of depression significantly enhances the risk of patients contracting other diseases, including cardiovascular diseases (Van der Kooy et al., 2007), stroke (Ramasubbu & Patten, 2003), Alzheimer's disease (Ramasubbu & Patten, 2003), diabetes (Mezuk et al., 2008), and more. The pathogenesis of depression is quite complex and is typically caused by multiple factors. Currently recognized pathogenic mechanisms include the reduction of central monoamine neurotransmitters or receptors, such as serotonin, dopamine, etc. (Bai et al., 2018), endocrine disorders caused by the overactivation of the hypothalamic–pituitary–adrenal (HPA) axis (Niemegeers et al., 2017), upregulated concentration levels of pro‐inflammatory factors, such as interleukin 6 (IL‐6), tumor necrosis factor‐α (TNF‐α), and interleukin 1 beta (IL‐1β) (Raison et al., 2006), imbalance in the synaptic cleft amino acid neurotransmitters, such as glutamate concentration, and reduced or obstructed clearance rates (Bian et al., 2023), and a decrease in the abundance of gut microbiota (such as families *Lachnospiraceae* and *Ruminococcaceae*) (Radjabzadeh et al., 2022). Currently, synthetic chemical drugs are the most usual and resultful means for depression treatment in clinical practice, but they have the shortcomings of low efficiency, severe side effects, and high costs (Carhart et al., 2021). However, as an alternative and complementary therapy, functional vegetable crops have unique benefits on the prevention and treatment of depression, with stable pharmacological effects, fewer adverse reactions, and high safety (Wang et al., 2022). Therefore, the research and development (R&D) of new drugs from functional vegetable crops have significant implications for depression treatment and the innovative medications’ development.

*Hemerocallis citrina* Baroni, *H*. *citrina*, is a plant belonging to the *Liliaceae* family, widely cultivated in China, Japan, and Korea, known for its delicious flavor and rich physiological effects with a history spanning thousands of years. The famous Chinese medical encyclopedia called “Compendium of Materia Medica” composed by Shizhen Li (1518–1593 AD) recorded that *H*. *citrina* had effects, such as improving sleep, preventing depression, promoting lactation, and aiding digestion (Liang, Wei, et al., 2023; Ma et al., 2023). The *H*. *citrina* flower buds have been used for thousands of years as a vegetable or medicinal plant. Recent pharmacological studies have indicated that the bioactive properties of the edible flowers of *H*. *citrina* include antioxidant (Wang et al., 2018), anti‐tumor (Sang et al., 2023), anti‐constipation (Liang, Wei, et al., 2023), anti‐depression (Jiang et al., 2023; Liu et al., 2022), treatment of insomnia (Liang, Zhan, et al., 2023; Zhong et al., 2021), and treatment of lactation deficiency (Guo et al., 2023; Zhong et al., 2021). Recently, the anti‐depressive functions of *H*. *citrina* edible flowers have increasingly garnered attention of many researchers. However, currently, many scholars are only studying the anti‐depressive‐like activity and mechanisms of the ethanol or aqueous extracts from the edible flowers of *H*. *citrina*. In preliminary research, Gu et al. and Yi et al. revealed that the ethanol *H*. *citrina* extractions (HCE) could enhance monoamines and brain‐derived neurotrophic factor (BDNF) levels in corticosterone (CORT)‐induced and acute stress‐induced depression‐like models of rodents, thereby exhibiting anti‐depressant‐like effects (Gu et al., 2012; Yi et al., 2012). Similarly, Li et al. also confirmed the anti‐depressive effects of HCE with a lipopolysaccharide (LPS)‐induced mice model of depression, resulting that HCE significantly reversed the decrease of sucrose preference in mice due to the activating expression of iNOS (inducible nitric oxide synthase), NF‐κB (nuclear factorkappa B), and COX‐2 (cyclooxygenase‐2) in the prefrontal cortex by LPS (Li et al., 2017). In recent years, some scholars have also suggested that the anti‐depression‐like actions of *H*. *citrina* extracts may be related to gut microbiota. Liu et al. discovered that the water extract of *H*. *citrina* flowers (HCW) exerted their anti‐depressive activity by enhancing the abundance of *Bacteroides* genera, thereby improving the level of GABA (gamma‐aminobutyric acid) (Liu et al., 2022). HCW could also significantly downregulate the levels of the *Desulfovibrio* genera in the gut, blood, and brain of mice induced by depressed models, diminishing the content of inflammatory factors, thus contributing to its anti‐depressive effect (Liu et al., 2022). However, the specific and accurate material basis and the molecular actions on anti‐depressive effects for *H*. *citrina* flowers are not yet fully understood.

Network pharmacology integrates research fields, such as genomics, proteomics, bioinformatics, and pharmacology, allowing for a systematic description of the interrelationships between traditional Chinese medicine (TCM) and its compounds with diseases. Network pharmacology studies multiple components, targets, and pathways to explore the biological mechanisms by which constituents process their activities (Chen et al., 2023; Liu et al., 2023). Therefore, network pharmacology is widely applied in the screening for potentially active compounds in TCM, predicting TCM or disease targets, and interpreting the fundamental pharmacological actions of drugs on diseases and their specific mechanisms (Zhao et al., 2023). For example, Liang et al. investigated the molecular basis and mechanism of *H. citrina* in improving sleeping based on the network pharmacology with experimental validation using *Drosophila* activity monitoring and real‐time quantitative polymerase chain reaction (q‐PCR), revealing that quercetin, phenethyl caffeate, linoleic acid, L‐methionine, and γ‐aminobutyric acid were the core active compounds and that the neuroactive ligand–receptor interaction was the key pathway to modulate the sleep‐improving effects (Liang, Zhan, et al., 2023). Based on the ligand–receptor interaction theory, molecular docking methods are widely used to understand how compounds bind with the primary molecular targets, which plays a key role in drug discovery (Mir et al., 2023). In our study, the network pharmacology studies were employed to screen the potential anti‐depressive active components in *H*. *citrina* edible flower parts and their core anti‐depression targets. Molecular docking method was then used to prove the interaction between central targets and major active components. Finally, based on in vitro experimental analysis, the molecular mechanisms of the most effective bioactive components of *H*. *citrina* flowers and the key therapeutic targets for alleviating depression were discussed.

## 2 MATERIALS AND METHODS

### 2.1 Screening of active compounds and targets of *H*. *citrina* edible flower

Active compounds of the *H*. *citrina* edible flower (HEF) were preliminarily obtained from HERB (a high‐throughput experiment and reference‐guided database of TCM) database and Google Scholar, with *Hemerocallis citrina*, flower, metabolites, and edible as keywords. After deduplicating the results from both ways, the main active compounds of HEF were selected based on the DL (drug‐likeness) ≥0.18 and OB (oral bioavailability) ≥30% (Lin et al., 2020). The structures and canonical SMILES (Simplified Molecular‐Input Line‐Entry System) of all major active compounds were prepared using the PubChem database (https://pubchem.ncbi.nlm.nih.gov/, accessed on September 1, 2023) (Kim et al., 2023) or obtained from confirmed literature. ChemDraw 2D software was constructed for drawing the two‐dimensional (2D) structures of the main compounds. To identify the targets of active compounds in HEF, the similarity ensemble approach (SEA) database (https://sea.bkslab.org/, accessed on September 5, 2023) (Keiser et al., 2007), SwissTargetPrediction database (http://www.swisstargetprediction.ch/, accessed on September, 5 2023) (Daina et al., 2019), and TCMSP (the traditional Chinese medicine systems pharmacology database and analysis platform) database (https://tcmsp‐e.com, accessed on September 5, 2023) (Yang et al., 2013) were used, with “*Homo sapiens*” as species selection to obtain human gene targets. All retrieved protein targets were imported into the UniProt (The Universal Protein Resource) database (https://www.uniprot.org/, accessed on September 5, 2023) (Consortium, 2022) to obtain the corresponding gene names of the targets. The final targets of HEF were determined after the deduplication of results from the three above‐mentioned databases.

### 2.2 Screening of depression‐related targets and potential anti‐depression targets for *H*. *citrina* edible flower

Gene targets associated with depression‐related disorders were obtained from the following four databases, filtering for “*Homo sapiens*” or “*Human*” to identify human‐derived genes. Using “depression” as a keyword, we used the DrugBank database (https://www.drugbank.com, accessed on September 10, 2023) (Wishart et al., 2018) to obtain the corresponding protein target names as well as their UniProt IDs of all drugs related to depression treatment. To get the corresponding gene targets of above‐mentioned proteins, we input the UniProt IDs into UniProt database, ultimately obtaining all depression‐related gene targets from the DrugBank database. The keyword “depression” was input into the GeneCards database (https://www.genecards.org/, accessed on September 10, 2023) (Stelzer et al., 2016), and gene targets categorized as “Protein Coding” were selected out. We ultimately screened all the targets whose GIFTS (GeneCards Inferred Functionality Score) were at or below the median value as the final depression‐related targets from the GeneCards database. We also used the DisGeNet database (http://www.disgenet.org/, accessed on September 10, 2023) (Piñero et al., 2015) to obtain the depression‐related targets, with the keywords named depressive disorder, mental depression, major depressive disorder, unipolar depression, mixed anxiety and depressive disorder, depression, bipolar, depressive disorder, treatment‐resistant, depression, postpartum, endogenous depression, severe depression, depressive syndrome, major depression, single episode, depression, neurotic, depressed bipolar I disorder, recurrent major depressive episodes, recurrent depressive disorder, depressive episode, unspecified, involutional depression, recurrent depression, perinatal depression in mother, depression and suicide, clinical depression, severe major depression with psychotic features, depression, psychotic, winter depression, depression in children, mild depression, atypical depressive disorder, circulatory depression, chronic depression, menopausal depression, acute depression, severe major depression, melancholic depression, and drug‐induced depression. Using “major depressive disorder” as a keyword, the OMIM (Online Mendelian Inheritance in Man) database (https://www.omim.org/, accessed on September 11, 2023) (McKusick, 2009) was also used to identify targets related to depression. The gene targets obtained from the four databases were merged and deduplicated to get the final gene targets related to depression. We used the online tool, Venny (https://bioinfogp.cnb.csic.es/tools/venny/, accessed on September 11, 2023) (Oliveros, 2007), to perform an intersection analysis and draw a Venn diagram of the predicted gene targets of the main active compounds in HEF and the gene targets related to depression. The intersecting gene targets of HEF and depression served as potential anti‐depression targets.

### 2.3 Core targets screening and protein–protein interaction (PPI) network construction

The potential anti‐depression targets of HEF were imported into the STRING (Search Tool for the Retrieval of Interacting Genes/Proteins) platform (https://stringdb.org/, accessed on September 14, 2023) (Szklarczyk et al., 2023), with setting the organism to “*Homo sapiens*” and the minimum required interaction score to “0.4”, yielding a PPI map of the potential anti‐depression targets of HEF. For visualization analysis, we selected “enable 3D bubble design” to obtain a three‐dimensional (3D) PPI map and downloaded the Tab‐Separated Values (TSV) format data file of the interaction network results. To further filter the core targets related to depression, TSV format data file exported from the STRING platform was analyzed using Cytoscape 3.9.1 software (Shannon et al., 2003). In the CytoNCA of Cytoscape, the degree centrality (DC), closeness centrality (CC), and betweenness centrality (BC) were employed to identify corresponding targets. Using Venny online tool, the targets whose topological parameters were all higher than the median value of the three parameters were selected for intersection analysis. The final intersecting targets were the core anti‐depression targets of HEF (Jiao et al., 2023; Liu et al., 2023). For visualization of the degree values of targets, in Cytoscape software, the node color was adjusted according to DC to, respectively, obtain the PPI maps for the potential anti‐depression targets of HEF, and for the core anti‐depression targets.

### 2.4 Drug‐compounds–targets‐disease network construction

The intersection analysis between the gene targets corresponding to each screened active compound of HEF and the core targets was carried out using online Venny tool, obtaining the intersection targets, which are the core anti‐depression targets of each active compound. The core anti‐depression targets of each active compound were merged and deduplicated to obtain all the targets required for the network diagram. Two files in Excel, a “network” file, and a “type” file, were created, including the main active compound names, the core anti‐depression targets of HEF, the core anti‐depression targets for each active compound in HEF, disease name, drug names, etc., and were applied for constructing the drug‐compounds–targets‐disease network by Cytoscape 3.9.1.

### 2.5 Gene Ontology (GO) and Kyoto Encyclopedia of Genes and Genomes (KEGG) enrichment analysis of core targets

The GO enrichment analysis and KEGG enrichment analysis for core anti‐depression targets of HEF were obtained through the Metascape database (https://metascape.org/gp/index.html#/main/step1, accessed on September 25, 2023) (Zhou et al., 2019). The GO enrichment analysis contained three parts, biological processes (BPs), CC, and molecular functions (MFs). To perform the GO enrichment analysis and KEGG enrichment analysis, we imported the potential anti‐depression targets of HEF into the Metascape database and limited the organism to “*Homo sapiens*” and set the cutoff *P value* to *0*.*05*, the minimum overlap to 3, and the minimum enrichment to 1.5. Bubble diagrams for the GO enrichment analysis and KEGG enrichment analysis of the core targets were plotted by https://www.bioinformatics.com.cn (accessed on October 11, 2023) for data analysis and visualization.

### 2.6 Central targets screening and molecular docking

Meanwhile, for the final molecular docking, we used the CytoHubba of Cytoscape software to analyze the PPI network results exported from the STRING platform to obtain the top 10 central targets based on DC, CC, maximum neighborhood component (MNC), and maximal clique centrality (MCC) (Liu et al., 2023). The online Venny tool was used to perform intersection analysis on the gene targets corresponding to the four parameter values and obtain the intersecting targets, which were the central anti‐depression targets of HEF. Molecular docking is a technical method that simulates the interaction between ligand small molecules and receptor biomacromolecules based on the principle of complementarity in shape and properties of the ligand and receptor (Du et al., 2023). We performed the molecular docking on central anti‐depression targets of HEF and the top 10 active compounds ranked by degree in the Drug‐Compounds–Targets‐Disease network. The 3D structure of each selected active compound was obtained using ChemDraw 3D software and saved as Protein Data Bank (PDB) format files. After literature search and resolution filtering (<2), we downloaded the structure of each central target from the Research Collaboratory for Structural Bioinformatics Protein Data Bank (RCSB PDB) database (https://www.pdbus.org, accessed on October 20, 2023) (Berman et al., 2000) and also saved as PDB format files. The receptor files (central target) and the ligand 3D files (active compound) were converted into pdbqt format with the water removal or hydrogens’ addition using AutoDockTools 1.5.7 software (Liu et al., 2023). After restarting AutoDockTools 1.5.7, the prepared pdbqt files of the receptor and ligand were imported. By adjusting the docking parameters, we built a docking box with the receptor protein at the center, which means the receptor protein should be covered by the docking box completely, leading the ligand to locate outside the docking box. Subsequently, the docking box parameters were collected, and molecular docking was applied through AutoDock Vina 1.1.2, where the magnitude of the binding energy reflects the likelihood of receptor–ligand binding. The lower the binding energy, the higher the affinity between the receptor and ligand, which also means a more stable conformation of the receptor and ligand (He et al., 2023). Matrix Heatmap for molecular docking results was plotted by https://www.bioinformatics.com.cn (accessed on October 24, 2023), an online platform for visualization analysis. PyMOL 2.5.0 and Discovery Studio software were used to visualize the molecular docking results.

## 3 RESULTS

### 3.1 Active compounds and targets of *H*. *citrina* edible flowers

After screening the literature and database results, 830 active compounds were identified in *H*. *citrina* edible flowers, including flavonoids, phenylpropanoids, phenolic acids, etc. After filtering through OB and DL, 24 main active compounds were selected. The names, PubChem IDs, OB and DL values, canonical SMILES, and 2D structural diagrams of these 24 compounds are shown in Table 1. After comprehensive screening by databases mentioned before and deduplicating, a total of 614 potential targets of HEF were acquired.

**TABLE 1:** Twenty‐four active compounds and their parameter information of *H*. *citrina*.

Pubchem ID	Chemical name	2D structure	OB	DL	Anti‐depression target number	Reference
439533	Taxifolin	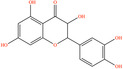	57.84	0.27	40	Liang et al. (2021), Liang, Zhan, et al. (2023), Zhong et al. (2021)
5280863	Kaempferol	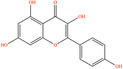	41.88	0.24	180	Hao et al. (2022), Liang et al. (2021), Liang, Zhan, et al. (2023) Ma et al. (2022), Zhong et al. (2021)
5280445	Luteolin	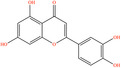	36.16	0.25	173	Liang et al. (2021)
440735	Eriodictyol	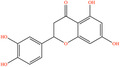	71.79	0.24	102	Liang et al. (2021), Liang, Zhan, et al. (2023), Zhong et al. (2021)
5281670	Morin	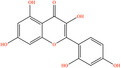	46.23	0.27	146	Liang et al. (2021), Zhong et al. (2021)
5280343	Quercetin	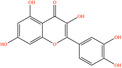	46.43	0.28	230	Hao et al. (2022), Liang et al. (2021); Liang, Zhan, et al. (2023), Ma et al. (2022), Zhong et al. (2021)
5489605	Demethylwedelolactone	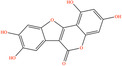	72.13	0.43	99	Liang et al. (2021), Zhong et al. (2021)
5379033	Dehydrodiisoeugenol	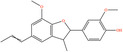	56.84	0.29	201	Liang et al. (2021), Zhong et al. (2021)
5280544	Herbacetin	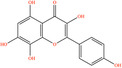	36.07	0.27	140	(Zhong et al., 2021)
72281	Hesperetin	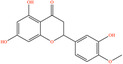	70.31	0.27	116	Hao et al. (2022), Liang, Zhan, et al. (2023)
439246	Naringenin	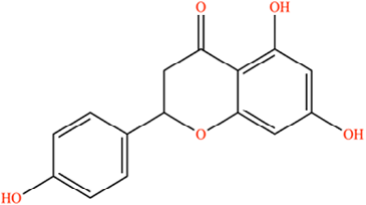	59.29	0.21	136	Hao et al. (2022), Liang, Zhan, et al. (2023)
5281654	Isorhamnetin	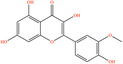	49.6	0.31	169	Hao et al. (2022), Liang, Zhan, et al. (2023), Ma et al. (2022)
5281612	Diosmetin	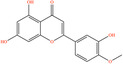	31.14	0.27	146	Hao et al. (2022), Liang, Zhan, et al. (2023)
9945785	3‐p‐Coumaroylquinic acid	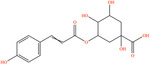	37.63	0.29	52	Liang, Zhan, et al. (2023), Ma et al. (2022)
25175592	3‐O‐Feruloylquinic acid	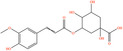	25.51	0.36	106	Liang, Zhan, et al., (2023), Ma et al. (2022)
64971	Betulinic acid	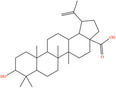	55.38	0.78	59	Liang, Zhan, et al. (2023)
6474640	Cynarine	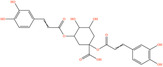	31.76	0.68	47	Liang, Zhan, et al. (2023)
28125525	Butin	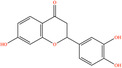	69.94	0.21	112	Liang, Zhan, et al. (2023)
5281628	Hispidulin	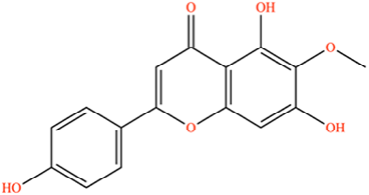	30.97	0.27	142	Liang, Zhan, et al. (2023)
5281,699	Tamarixetin	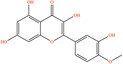	32.86	0.31	149	Liang, Zhan, et al. (2023)
5281678	Patuletin	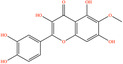	53.11	0.34	139	Liang, Zhan, et al. (2023)
72344	Nobiletin	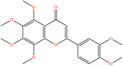	61.67	0.52	73	Liang, Zhan, et al. (2023)
67128659	Luteolin‐4’‐O‐glucoside	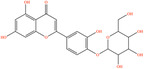	35.94	0.79	77	Liang, Zhan, et al. (2023)
5351516	Ergosterol peroxide	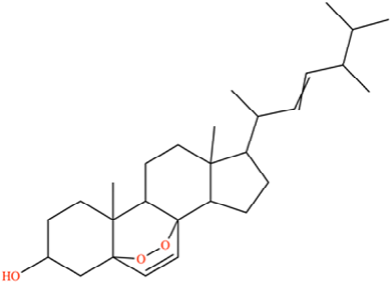	42.84	0.74	51	Liang, Zhan, et al. (2023)

### 3.2 Depression‐related targets and potential anti‐depression targets for *H*. *citrina* edible flowers

**FIGURE 1:**
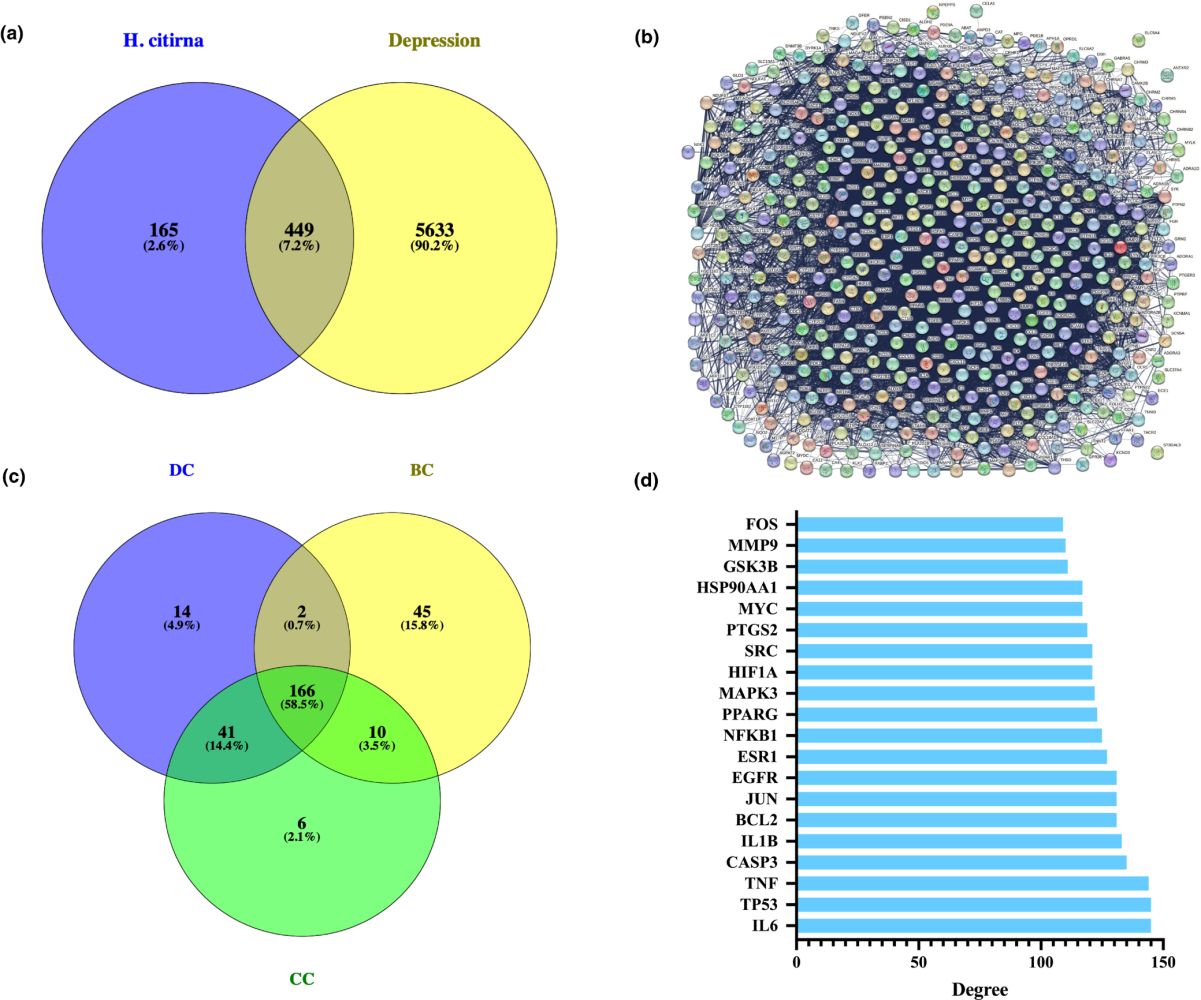
Screening of core anti‐depression targets in HEF. (a) Venny diagram showing potential anti‐depression targets. (b) PPI network of 449 potential anti‐depression targets by STRING. (c) Venny diagram showing core potential anti‐depression targets. (d) Top 20 potential anti‐depression targets ranked by degree values.

From the DrugBank database, 95 targets related to depression were obtained. For GeneCards database, a total of 10,031 depression‐related targets were obtained and after filtering through the median value in GIFTS, 5179 targets related to depression were finally obtained. The DisGeNet database did not have a comprehensive summary of the major category of depression but organized the diseases contained within the major category of depression one by one, and the number of targets corresponding to its minor diseases is shown in Table 2. After merging and filtering the targets corresponding to all depression‐related diseases, a total of 2496 depression‐related targets were obtained from the DisGeNet database. However, the OMIM database only had gene targets related to major depressive disorder, 6 in total. By deduplicating all the targets obtained from the above four databases, 6082 targets for depression were obtained. The Venn diagram drawn after the intersection analysis between the targets for main active components of HEF and the depression‐related targets is shown in Figure 1a, which showed that there were 449 potential anti‐depression targets of HEF.

**TABLE 2:** The detailed information of depressive‐related disease collected from DisGeNet database.

Disease name	CUI	Targets number
Depressive disorder	C0011581	1719
Mental depression	C0011570	1478
Major depressive disorder	C1269683	641
Mixed anxiety and depressive disorder	C0338908	146
Depression, bipolar	C0005587	116
Depressive disorder, treatment‐resistant	C2063866	66
Depression, postpartum	C0221074	54
Endogenous depression	C0011573	53
Severe depression	C0588008	46
Depressive syndrome	C0086133	45
Major depression, single episode	C0024517	42
Depression, neurotic	C0282126	41
Depressed bipolar I disorder	C0236773	37
Recurrent major depressive episode	C0154409	36
Recurrent depressive disorder	C0349218	29
Depressive episode, unspecified	C0349217	27
Involutional depression	C0011574	25
Recurrent depression	C0221480	19
Perinatal depression in mother	C4284586	15
Depression and suicide	C1524032	15
Clinical depression	C2362914	14
Severe major depression with psychotic features	C0270458	12
Depression, psychotic	C0743072	11
Winter depression	C0871610	9
Depression in children	C3826462	8
Mild depression	C0588006	8
Atypical depressive disorder	C0154437	7
Circulatory depression	C1610069	5
Chronic depression	C0581391	4
Menopausal depression	C0520665	3
Acute depression	C1386135	2
Severe major depression	C3472470	1
Melancholic depression	C0025193	51
Drug‐induced depression	C0338715	13

### 3.3 Core potential anti‐depression targets of *H*. *citrina* edible flowers and PPI network analysis

**FIGURE 2:**
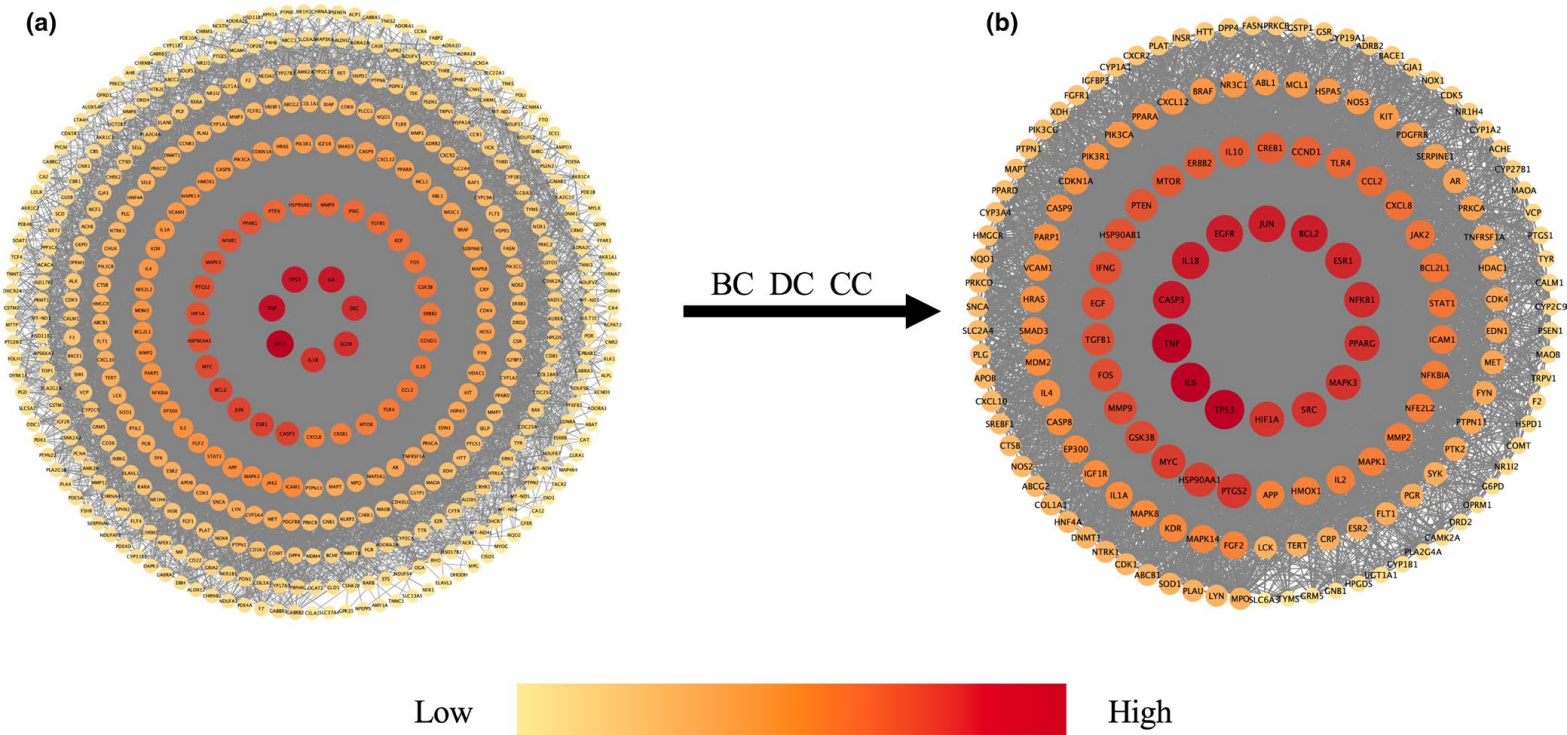
Protein–protein interaction (PPI) construction of core anti‐depression targets in HEF. (a) PPI network of 449 potential anti‐depression targets by Metascape. (b) PPI network of 166 core potential anti‐depression targets by Metascape. Darker the red color and larger nodes means higher degree of the target, and more targets interact with it. Conversely, lighter color and smaller nodes mean lower degree of the target, and fewer targets interact with it.

The PPI network of 449 potential anti‐depression targets from HEF, obtained using the STRING platform, is shown in Figure 1b. The analysis results of the interaction network using three parameters (DC, CC, and BC) from the CytoNCA are shown in the intersection Venn diagram (Figure 1c). The results indicated that there were 166 core anti‐depression targets of HEF in total. The PPI network of potential anti‐depression targets of HEF obtained using Metascape software is shown in Figure 2a, with a total of 444 nodes and 11,361 edges. Among them, based on the DC values, the top 20 genes, as shown in Figure 1d, revealed that TP53 and IL‐6 have the highest degree values, both at 145. After filtering through DC, CC, and BC, the PPI network of HEF's core anti‐depression targets is shown in Figure 2b, comprising 165 nodes and 5449 edges.

### 3.4 Drug‐compounds–targets‐disease network analysis

**FIGURE 3:**
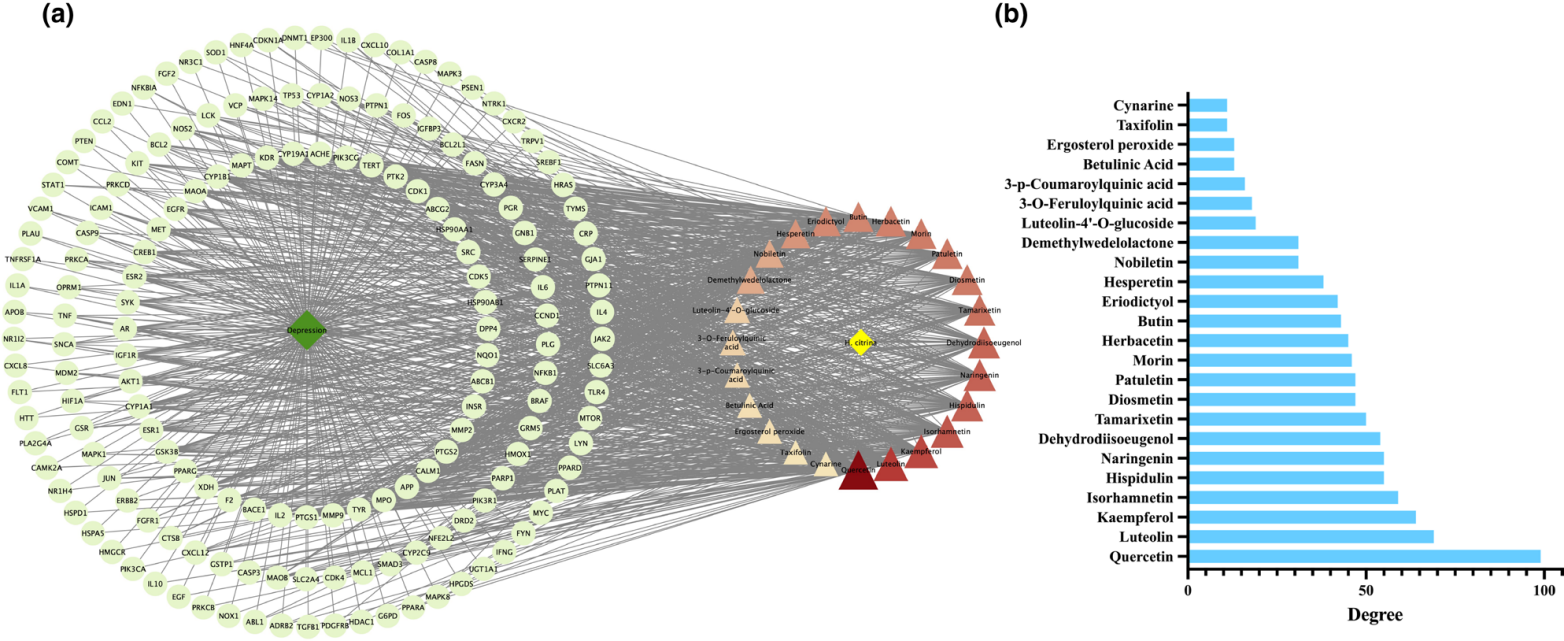
Drug‐compounds–Targets‐Disease Network Analysis. (a) Drug‐compounds–Targets‐Disease Network. Triangle nodes represent active compounds; ellipse nodes represent core potential anti‐depression targets; green diamond nodes represent the disease name; yellow diamond nodes represent the drug name. Larger triangle nodes and darker color of triangle nodes mean higher degree of active compounds, and more core anti‐depressant targets they correspond to. (b) Twenty‐four active compounds from HEF ranked by degree values.

Figure 3a illustrates that the drug‐compounds–targets‐disease network was composed of 191 nodes and 1141 edges, which included 24 active compounds, 165 core potential anti‐depression targets, one disease name, and one drug name. The ranking of the 24 active compounds in the drug‐compounds–targets‐disease network by degree value is shown in Figure 3b, where the top 10 active compounds in descending order of degree were quercetin (99), luteolin (69), kaempferol (64), isorhamnetin (59), hispidulin (55), naringenin (55), dehydrodiisoeugenol (54), tamarixetin (50), diosmetin (47), and patuletin (47).

### 3.5 GO enrichment and KEGG enrichment analysis of core targets

**FIGURE 4:**
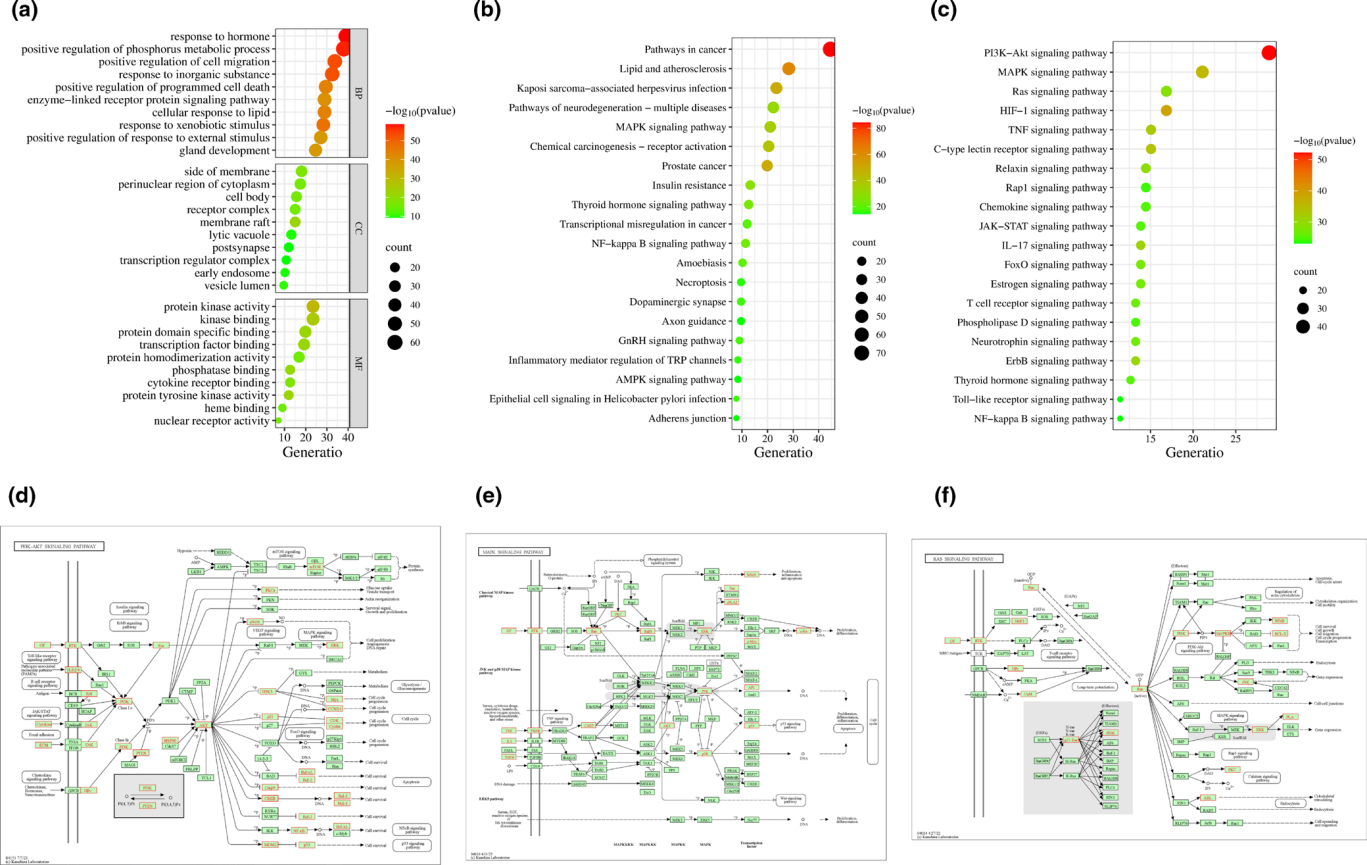
KEGG and GO enrichment analysis of 166 core anti‐depression targets in HEF. (a) Bubble diagram of the top 20 KEGG pathways. (b) Bubble diagram of the top 10 GO BPs, GO CCs, and GO MFs. (c) Bubble diagram of the top 20 signaling pathways. (d) PI3K‐Akt signaling pathway (hsa04151). (e) MAPK signaling pathway (hsa04010). (f) Ras signaling pathway (hsa04014).

The KEGG enrichment and GO enrichment analysis were conducted to study the biological processes and metabolic pathways potentially involved in the anti‐depression effects of HEF. Regarding the GO results (Figure 4b), GO BPs enrichment analysis mainly involved negative regulation of osteoclast differentiation, positive regulation of phosphorus metabolic process, positive regulation of cell migration, response to inorganic substance, and positive regulation of programmed cell death, among others. GO CCs enrichment analysis indicated that the side of membrane, perinuclear region of cytoplasm, cell body, receptor complex, and membrane raft were the main cellular localizations involved in anti‐depression. GO MFs enrichment analysis revealed that protein kinase activity, kinase binding, protein domain‐specific binding, transcription factor binding, and protein homodimerization activity were the primary molecular functions (MFs) involved in anti‐depression of HEF. From the KEGG results (Figure 4a), we found that KEGG pathways were mainly enriched in pathways in cancer (hsa05200), lipid and atherosclerosis (hsa05417), kaposi sarcoma‐associated herpesvirus (KSHV) infection (hsa05167), pathways of neurodegeneration–multiple diseases (hsa05022) and mitogen‐activated protein kinase (MAPK) signaling pathway (hsa04010). In addition, after extracting all signaling pathways from KEGG results (Figure 4c), we found that PI3K‐Akt signaling pathway (hsa04151) (Figure 4d), MAPK signaling pathway (hsa04010) (Figure 4e), and Ras signaling pathway (hsa04014) (Figure 4f) were the three enriched signaling pathways.

### 3.6 Central targets screening

**FIGURE 5:**
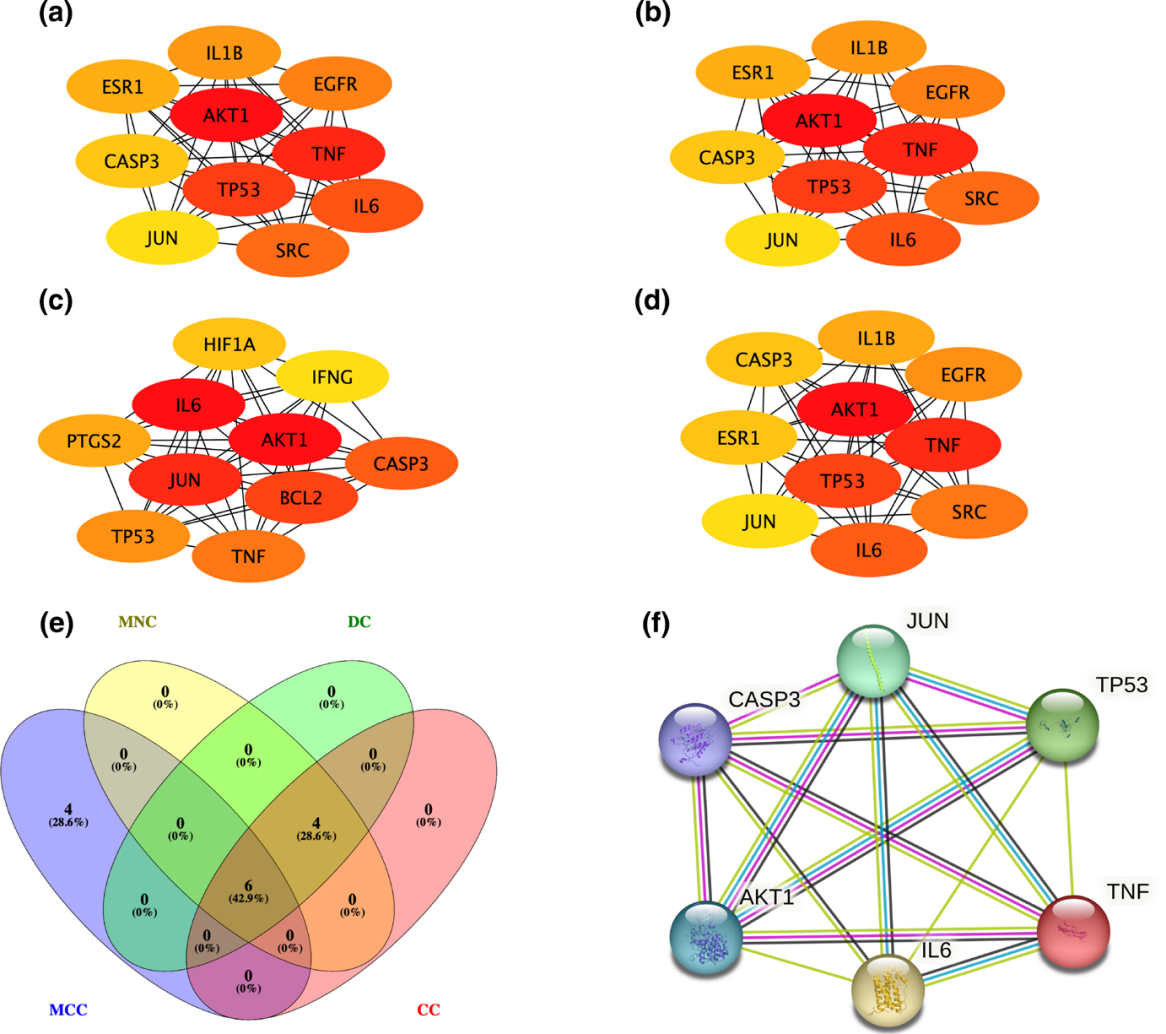
The network of top 10 core anti‐depression targets was screened using (a) MNC, (b) Degree, (c) MCC, and (d) closeness. Darker red color of the nodes means higher degree of the targets. (e) Venn diagram showing six central anti‐depression targets. (f) PPI network of six central anti‐depression targets by STRING.

Top 10 core anti‐depression targets screened using MNC or CC were both AKT serine/threonine kinase 1 (AKT1), TNF, TP53, IL‐6, SRC, epidermal growth factor receptor (EGFR), IL‐1β, estrogen receptor 1 (ESR1), caspase 3 (CASP3), and JUN (Figure 5a and Figure 5d). Top 10 core anti‐depression targets screened using DC were AKT1, TNF, TP53, IL‐6, SRC, EGFR, IL‐1β, CASP3, ESR1, and JUN (Figure 5b). Top 10 core anti‐depression targets screened using MCC were IL‐6, AKT1, JUN, B‐cell lymphoma 2 (BCL2), CASP3, TNF, TP53, prostaglandin G/H synthase‐2 (PTGS2), hypoxia‐inducible factor subunit alpha 1 [(human)] (HIF1A), and interferon gamma (IFN‐γ) (Figure 5c). The intersection by Venny of core targets obtained from these four screening results were the central targets (Figure 5e). Therefore, the central targets were IL‐6, AKT1, JUN, CASP3, TNF, and TP53 (Figure 5f).

### 3.7 Molecular docking

**FIGURE 6:**
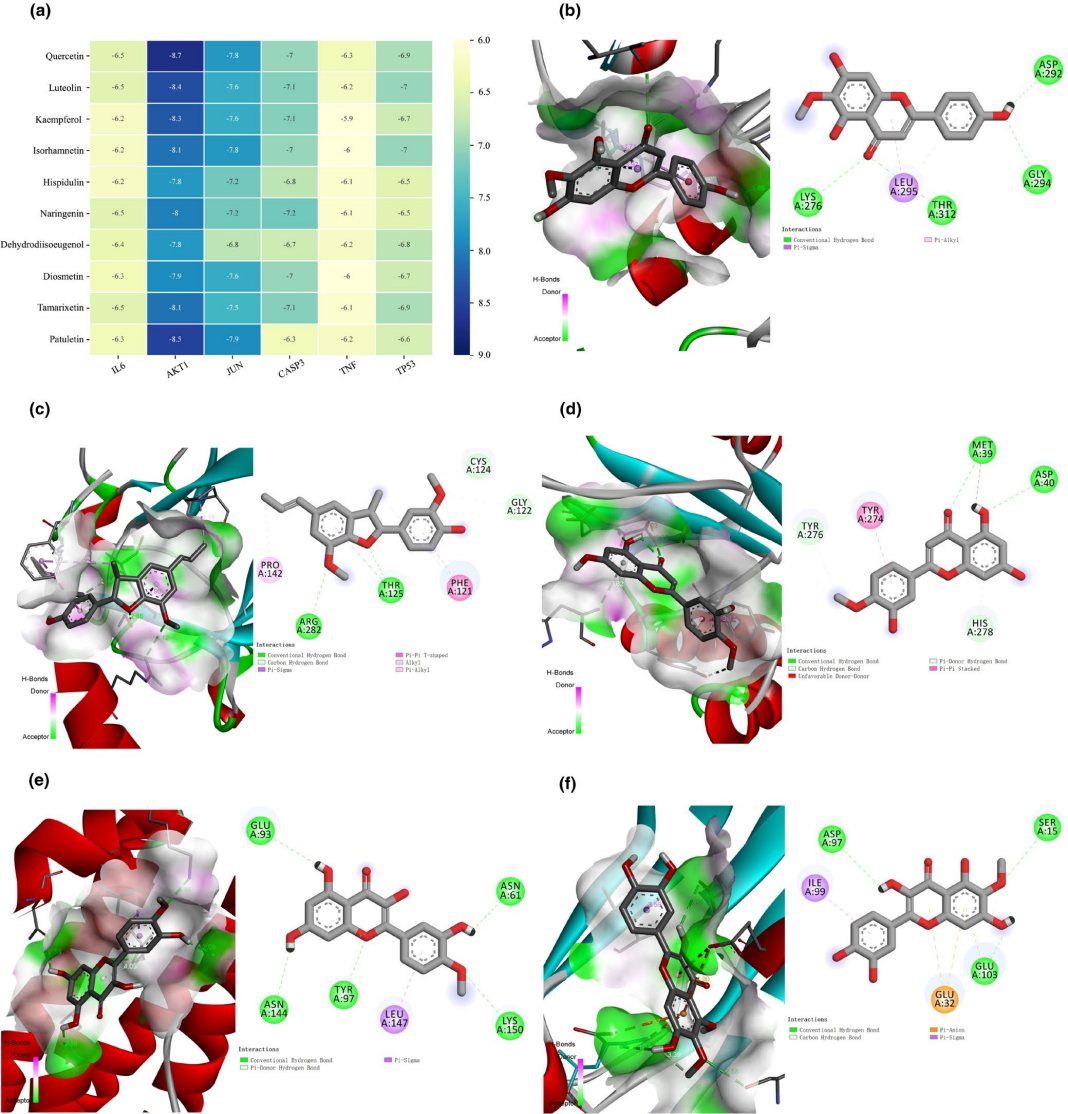
Molecular docking results. (a) Matrix Heatmap for molecular docking scores (kcal·mol^−1^) between central targets and top 10 anti‐depressant active compounds ranked by degree. Lower value and darker color indicate a stronger binding ability. Molecular docking between hispidulin and AKT1 (b), dehydrodiisoeugenol and TP53 (c), diosmetin and CASP3 (d), tamarixetin and IL‐6 (e), and patuletin and TNF (f).

Using the central targets as receptors and the top 10 active compounds from HEF by degree as ligands, a docking process was conducted to obtain the binding energy between the ligands and receptors. A lower binding energy indicated a lower energy state and a more stable conformation. The top 10 active compounds included quercetin, luteolin, kaempferol, isorhamnetin, hispidulin, naringenin, dehydrodiisoeugenol, tamarixetin, diosmetin, and patuletin. The central targets included IL‐6 (PDB ID: 1ALU) (Aher et al., 2020), AKT1 (PDB ID: 4GV1) (Sharif Siam et al., 2021), JUN (PDB ID: 1PXZ) (Lai et al., 2022), CASP3 (PDB ID: 2J30) (Cade et al., 2014), TNF (PDB ID: 4G4F) (Lai et al., 2022), and TP53 (PDB ID: 4MZI) (Lai et al., 2022). The heatmap of the molecular docking results (Figure 6a) indicated that the binding energy values between these 10 effective components from HEF and the central targets were all below −6.0 kcal/mol. It is generally believed that a binding energy of <−5.0 kcal/mol indicates a good binding effect. All docking results in our study were less than 0 and even less than −5.0 kcal/mol, showing that the main compounds could bind well to the central targets with stable conformation. Among them, IL‐6 had the lowest binding energy with tamarixetin (−6.5 kcal/mol) and naringenin (−6.5 kcal/mol). AKT1 and quercetin combine to have the lowest binding energy, which is −8.7 kcal/mol. The lowest binding energy, with the combination of JUN and patuletin, was −7.9 kcal/mol. CASP3 had the lowest binding energy with naringenin (−7.2 kcal/mol). TNF and quercetin combine to have the lowest binding energy, −6.3 kcal/mol. TP53 had the lowest energy, −7 kcal/mol, binding with luteolin. PyMOL 2.5.0 and Discovery Studio software were used to visualize some of the docking complexes. Figure 6b illustrates that the docking interactions between hispidulin and AKT1 included conventional hydrogen bond, Pi–alkyl, and Pi–sigma. The interactions between dehydrodiisoeugenol and TP53 are shown in Figure 6c, including conventional hydrogen bond, carbon hydrogen bond, T‐shaped Pi–Pi, Pi–alkyl, alkyl, and Pi–sigma. The docking results of diosmetin with CASP3 are shown in Figure 6d and included conventional hydrogen bond, carbon hydrogen bond, Pi–donor hydrogen bond, Pi–Pi‐stacked, and unfavorable donor–donor interactions. Figure 6e displays the interactions between tamarixetin and IL6, which comprised conventional hydrogen bond, Pi–donor hydrogen, and Pi–sigma. Lastly, the interactions between patuletin and TNF are shown in Figure 6f, including conventional hydrogen bond, carbon hydrogen bond, Pi–anion, and Pi–sigma.

## 4 DISCUSSION

Within the “drug‐compounds‐targets‐disease” network, the top 10 compounds considered as potential therapeutic agents for depression were identified (quercetin, luteolin, kaempferol, isorhamnetin, hispidulin, naringenin, dehydrodiisoeugenol, tamarixetin, diosmetin, and patuletin). Among these, all except dehydrodiisoeugenol, which belongs to phenolic acid, are flavonoids. It has been reported that the anti‐depressive effects of hydroalcoholic extracts from *H*. *citrina* are mainly related to flavonoids (Du et al., 2014). Quercetin is a natural plant‐derived flavonoid compound with a broad spectrum of biological activities, commonly found in vegetables and fruits (Silvestro et al., 2021). Donoso et al. (Donoso et al., 2020) have demonstrated that quercetin could increase the plasma BDNF levels and ameliorate the HPA axis dysregulation, decreasing corticosterone plasma levels, which were related to the protection from depressive behaviors caused by maternal separation. Luteolin is a natural flavonoid, and current literature indicated that it had anti‐depressive functions, which were partly due to the inhibition of endoplasmic reticulum (ER) stress (Guan & Liu, 2016). For instance, Ishisaka et al. (Ishisaka et al., 2011) reported that in some behavioral tests, such as forced swimming test (FST) and tail suspension test (TST), chronic oral administration of luteolin exhibited an anti‐depressant‐like effect by decreasing the expression of stress‐related proteins in the hippocampus endoplasmic reticulum of mice with depression treated with CORT. Kaempferol is a flavonoid compound, widely found in various natural plants, fruits, and vegetables, such as tea, strawberries, apples, broccoli, green beans, and spinach (Ekici et al., 2023). Park et al. reported the anti‐depressant‐like activity of kaempferol isolated from *Opuntia ficus indica* var. *saboten* using TST and FST in chronic restraint stress (CRS) group of mice, suggesting that kaempferol could increase proopiomelanocortin messenger RNA (*POMC* mRNA) levels or plasma *β*‐endorphin levels and significantly reduce resting time in FST and TST, displaying a potent anti‐depressant effect (Park et al., 2010). Isorhamnetin is a kind of flavonoid observed in several plant‐based foods including grapes and apples, as well as in herbs such as *Ginkgo biloba* and sea buckthorn (Rammal et al., 2008). Ekici et al. (Ekici et al., 2023) studied the effects of isorhamnetin on depressive mice model induced by LPS through FST and TST, and the results demonstrated that isorhamnetin exerted an anti‐depressive action by promoting the prefrontal and hippocampal inflammation induced by LPS through the reduction of TNF‐α, IL‐1β, and IL‐6. All these findings supported the top four active compounds (quercetin, luteolin, kaempferol, and isorhamnetin) identified through the “drug‐compounds‐targets‐disease” network that had a potential ability for treating depression. Besides the top four core active compounds, naringenin, a potent anti‐inflammatory, antioxidant, and neuroprotective bioflavonoid, has also been indicated to possess anti‐depressant‐like activity (Olugbemide et al., 2021). The study by Olugbemide et al. (Olugbemide et al., 2021) suggested that naringenin ameliorated anxiety symptoms and depressive‐like behaviors stimulated by repeated hypoxic stress in mice partly through oxidative inflammatory response and positive regulation of the NF‐κB/BDNF pathway. Overall, according to the literature, there were many reports validating the anti‐depressant effects of the above five compounds, particularly quercetin. However, for other main identified active compounds, there were few direct reports of their anti‐depressant effects. For example, studies have shown that diosmetin could mitigate memory impairment caused by chronic unpredictable mild stress (CUMS) associated with neurological diseases (Saghaei et al., 2020), but there were hardly any studies proving the anti‐depressant effects of diosmetin. For the remaining active compounds such as hispidulin, dehydrodiisoeugenol, tamarixetin, and patuletin, there was almost no literature on their anti‐depressant effects or even related diseases. Therefore, future research on these remaining compounds may be of great importance.

The KEGG analysis consolidates data pertaining to chemical molecules and biochemical systems, encompassing metabolic pathways, drugs, diseases, gene sequences, and genomes, thus enabling the study of genes and expression information to form an interconnected network. It plays a key role in network pharmacology study, providing valuable information for therapeutic strategies and drug development. In accordance with the results from KEGG enrichment analysis, we identified that the core targets were widely distributed in the PI3K‐Akt signaling pathway, MAPK signaling pathway, and Ras signaling pathway. PI3K‐Akt signaling pathway, which most core targets enriched in, regulates cell survival, apoptosis, and differentiation (Tian et al., 2023) and plays an important role in the occurrence and development of diabetes, cancer, and neurological diseases (Sun et al., 2021). The downstream of PI3K/AKT pathway includes caspase 9 (CASP9), glycogen synthase kinase 3 (GSK3), protein 21 (P21), mammalian target of rapamycin (mTOR), and other pathways (Figure 4d), which have been widely reported to play a regulatory role in inflammatory response, depression, and cancer development (Bai et al., 2020; Ren et al., 2021). For example, the study of Tian et al. confirmed that the mRNA levels of *mTOR* decreased and *GSK‐3β* and forkhead box class O 3a (*FoxO3a*) increased in CUMS‐induced mice model, and regulation of the above gene targets could have a good effect against depression (Tian et al., 2023). Mitogen‐activated protein kinase (MAPK) has been supported by increasing evidence that it plays a key role in the symptomatology, pathogenesis, and treatment of depression, especially the extracellular signal‐regulated kinase (ERK) subclass of MAPK (Wang & Mao, 2019). In addition, four stress‐induced depression models, including CRS, CUMS, learned helplessness (LH), and social defeat (SD), in the hippocampus of rats, were analyzed metabolically and proteomically by Li et al., and the results identified by western blot showed that the above models mainly involved the related proteins (ERK) in the MAPK signaling pathway (Li et al., 2023). These studies suggested that modulating the key targets in these above signaling pathways could achieve the anti‐depressant effects, leading to promote the medicine development based on these therapeutic targets for depression.

We identified IL‐6, AKT1, JUN, CASP3, TNF, and TP53 as the central gene targets involved in depression treatment for *H*. *citrina* edible flower. In our study, interleukin 6 (IL‐6) is a hub target of anti‐depressant compounds in HEF. Its involvement as a pro‐inflammatory cytokine in the pathophysiology of depression has been confirmed by studies on human patients (Lindqvist et al., 2009) and corresponding animal models (Monje et al., 2011; Sukoff Rizzo et al., 2012). For example, Monje et al. found that IL‐6 knockout mice were resistant to the occurrence of depression‐like behavioral phenotypes after the constant darkness exposure, a chronobiology model of depression induced in mice, further supporting a functional role for IL‐6 in the molecular mechanisms of depression (Monje et al., 2011). Tumor protein 53 (TP53), as another hub target in HEF, is a transcription factor, participating in the regulation of gene expression involved in various cellular processes, including cell cycle, death, senescence, and DNA repair (Thomas et al., 2022). Epidemiological studies have indicated that TP53 was particularly involved in the regulation of MAPK signaling pathway, which was associated with the occurrence and development of major depressive disorder (MDD) (Chen et al., 2022). The serine–threonine protein kinase AKT1, as the most stable binding target to the 10 anti‐depressant compounds in HEF during molecular docking process, plays a crucial part in the regulation of biological functions, consisting of metabolism and cell proliferation (Androulidaki et al., 2009). Its polymorphism has been found to correlate with the severity of anxiety symptoms, depression, and tendency toward suicide in human patients (Yang et al., 2012). Tumor necrosis factor (TNF) is a core cytokine in mammals, initially identified for its capacity to induce necrosis in tumor cells (Sedger & McDermott, 2014). Subsequent findings, however, have revealed that tumor necrosis factor‐α (TNF‐α) was implicated not only in pathological processes such as immunity and inflammation but also played a critical role in physiological functions, especially within the central nervous system (CNS) (Uzzan & Azab, 2021). For example, studies have discovered that TNF‐α could damage the integrity of the blood–brain barrier (BBB) as an inflammatory factor (Cheng et al., 2018). The BBB dysfunction can accelerate inflammatory mediators and peripheral immune cells to the infiltration of the CNS, leading to depressive disorders and abnormal behaviors (Wohleb et al., 2011). In addition, other studies have revealed that plasma levels of TNF‐α and its soluble receptors were higher in patients with acute depression than those in normal individuals (Moisan et al., 2021). Caspase 3 (CASP3), as a key member of the cysteine proteinase caspase family, has been reported to be a major mediator of neuronal apoptosis in depressive patients (D'amelio et al., 2010). The investigation of Bliźniewska et al. (2023) showed that the expression of CASP3 in depressive patients was lower in both mRNA and protein levels than those in healthy groups, and CASP3 gene expression was positively correlated with the course of disease and the frequency of depressive episodes. Activated protein 1, shortened as AP‐1, is a key transcription factor during tumorigenesis, playing an important organic function in a wide variety of tumors (Chottekalapanda et al., 2020). Among them, c‐Jun, a key component of AP1, has been well demonstrated to be highly expressed in a variety of tumor‐related diseases and was regarded as a drug target for cancer therapy (Yu et al., 2022). Chottekalapanda et al. (2020) have found that AP‐1, including c‐Jun, could modulate the key neuronal remodeling gene expression, and its function was required for anti‐depressant actions in vivo. In summary, all six central targets from our findings have been identified as being associated with depression. Therefore, the identification of anti‐depression‐related targets can facilitate the study of potential anti‐depression mechanisms and is of great significance for the avoidance, treatment, and management of depression‐related diseases.

## 5 CONCLUSION

To conclude, our study utilized network pharmacology to determine the potential core targets and possible signaling pathways for the anti‐depressant effects of edible flower parts of *H*. *citrina*. IL‐6, AKT1, JUN, CASP3, TNF, and TP53 were named as central targets for the anti‐depressant effects of edible *H*. *citrina* flowers. Molecular docking analysis confirmed that the active components in *H*. *citrina* edible flowers had good affinity with the above targets. These above‐mentioned findings may promote the development of the *H*. *citrina* flower and its underlying use as a kind of therapeutic agent for depression‐related disorders. However, more pharmacological and clinical studies are needed to confirm our findings. In summary, our research has provided a framework for coming studies on the anti‐depressant mechanism of *H*. *citrina* flower and the application of network pharmacology for drug development.

## AUTHOR CONTRIBUTIONS

**Ruohan Zhao:** Data curation (equal); formal analysis (equal); investigation (equal); validation (equal); visualization (equal); writing – original draft (equal). **Jinhai Luo:** Formal analysis (equal); investigation (equal); methodology (equal); software (equal); validation (equal); writing – original draft (equal). **Sookja Kim Chung:** Conceptualization (equal); funding acquisition (equal); project administration (equal); validation (equal). **Baojun Xu:** Conceptualization (equal); funding acquisition (equal); project administration (equal); resources (equal); supervision (equal); validation (equal).

## FUNDING INFORMATION

This project was jointly supported by two grants (project code: UICR0400015‐24 and UICR0400016‐24) from BNU‐HKBU United International College.

## CONFLICT OF INTEREST STATEMENT

The authors declare no conflicts of interest.

## ETHICS STATEMENT

There are no experimental animals and human subjects involved in this study. Therefore, there is no ethical issue in this study.

## Data Availability

The data presented in this study are available on request from the corresponding author.
